# Survey of Colorado beef producers’ perceptions of the Beef Quality Assurance program

**DOI:** 10.1093/tas/txaf057

**Published:** 2025-04-29

**Authors:** Malei Tipton, Colton Smith, Paxton Sullivan, Tyler Thomas, Libby Bigler, Kayleigh Keller, Jason Ahola, Lily Edwards-Callaway

**Affiliations:** Department of Animal Sciences, Colorado State University, Fort Collins, Colorado 80523, USA; Department of Animal Sciences, Colorado State University, Fort Collins, Colorado 80523, USA; Department of Animal Sciences, Colorado State University, Fort Collins, Colorado 80523, USA; Department of Animal Sciences, Colorado State University, Fort Collins, Colorado 80523, USA; Department of Animal Sciences, Colorado State University, Fort Collins, Colorado 80523, USA; Department of Statistics, Colorado State University, Fort Collins, Colorado 80523, USA; Department of Animal Sciences, Colorado State University, Fort Collins, Colorado 80523, USA; Department of Animal Sciences, Colorado State University, Fort Collins, Colorado 80523, USA

**Keywords:** animal care, best management practices, BQA, cattle, producer

## Abstract

The Beef Quality Assurance (BQA) program was first established as a voluntary beef producer initiative to address food safety and quality challenges. A statewide survey of beef producers was conducted to determine: (1) prevalence of BQA certification status, (2) the relationship between BQA certification and adherence to best management practices, and (3) perceptions about the value of BQA certification. The survey was mailed to beef producers in Colorado and included questions about demographics, BQA knowledge and participation, perspectives on the BQA program, and value derived from BQA. A total of 466 producers were included in the analysis; nearly 95% (n = 439) had heard of the BQA program, and 72.0% (n = 313) had participated in BQA training. There was a significant relationship between BQA certification status and: perceived knowledge about and adherence to BQA guidelines (χ^2^ = 171.82, P < 0.0001), indication of having written documentation of a valid working relationship with a veterinarian (χ^2^ = 20.246, P < 0.0001), and following of animal handling and well-being practices (χ^2^ = 68.558, *P* < 0.0001). There was a relationship between being BQA-certification and using BQA certification as a marketing strategy (χ^2^ = 93.001, *P* < 0.0001). Results showed a relationship between BQA certification status and the belief that BQA certification increases consumer confidence in beef production (χ^2^ = 18.886, *P* < 0.0001). Results from this study indicate that producers identify many values associated with the BQA program related to cattle well-being, profitability, and consumer confidence.

## Teaser

Beef producers in Colorado value the Beef Quality Assurance (BQA) program for its benefits to animal welfare, profitability, and consumer confidence. This survey found associations between BQA certification and increased adherence to best management practices. BQA is an effective tool for improving cattle care.

## INTRODUCTION

The Beef Quality Assurance (BQA) program, originally called Beef Safety Assurance, was established by the National Cattlemen’s Beef Association (NCBA) in the late 1970s as a voluntary producer initiative to address food safety and meat quality challenges—most notably, residues and injection site lesions—and to improve consumer confidence in the beef they purchase (Klopatek et al., 2022). The BQA program has helped the beef industry make significant strides in improving meat quality, reducing economic losses, and promoting animal well-being. One notable achievement has been the consistent decline in the incidence of injection site lesions visible in carcasses, as evidenced by National Beef Quality Audits (NBQA) spanning from 1991 to 2022. Additionally, in the years since its inception, the BQA program—along with its suite of trainings, educational programming, and guidelines for best management practices—has evolved into a robust program that meets the changing needs of cattle producers and the broader beef industry.

In Colorado, the BQA program is uniquely positioned to serve a broad range of cattle producers, reflecting the diverse nature of the state’s beef industry. Not only is Colorado’s beef industry unique in that every sector of the supply chain is represented in the state (from the cow/calf producer to the packer), but also Colorado beef production spans multiple geographic regions, including operations from the western slope of the Rocky Mountains to the eastern plains. While the content of the BQA programming in Colorado may not differ significantly from that offered in other regions of the United States, the unique strength of BQA lies in its ability to tailor its delivery to various audiences across the state (personal communication, L. Bigler). The adaptability of the program and its associated training materials and resources allow BQA to cater to the specific needs and challenges faced by producers operating in different regions and industry sectors.

While some information is available regarding producers’ adherence to BQA guidelines, the studies have been conducted regionally and specific to one industry sector; for example, in the dairy industry (Gibbons-Burgener et al., 2000; Adams et al., 2016), on feedlots in the western United States (Woiwode et al., 2015, 2016), and on cow-calf operations in Montana (Duffey et al., 2008). In a nationwide survey of cattle ranchers, Klopatek and others (2022) characterized perceptions about BQA to gain insight into current involvement in BQA and motivations for adopting new production practices. The study reported that ranchers who were BQA-certified (currently or ever before) were more likely to administer health products following BQA guidelines compared to ranchers who had never been certified (Klopatek et al., 2022), highlighting the success of the program in promoting best practices for cattle care and health management. Additionally, the same survey reported that 80% of those surveyed shared that they believed the BQA program improved the health and welfare of cattle on their operations; this result reflects the emphasis that BQA has placed on championing responsible husbandry practices to advance animal well-being in the beef supply chain. While this nationwide survey provides valuable insights into the perceptions and practices of cattle ranchers regarding BQA certification, additional studies are necessary to formulate a robust understanding of the efficacy, reach, and impact of the program at a state level.

To address these gaps in knowledge, we conducted a statewide survey to investigate perceptions of cattle producers about the BQA program to better understand beef producer participation in, perceptions of, and opportunities to improve the CO BQA program. The objectives of this survey were to determine: (1) BQA certification status, (2) the relationship between BQA certification and adherence to best management practices, and (3) producer perceptions about the value of BQA certification.

## MATERIALS AND METHODS

Research protocols were reviewed prior to project initiation and deemed exempt by Colorado State University (CSU) Institutional Review Board (#3797).

### Study Population and Recruitment

A modified Dillman approach—a survey design method based on social exchange theory that aims to increase the response rate and accuracy of participants by increasing benefits and reducing risk from responding—was used as the framework for this survey (Dillman, 2007). Recipients were not offered an incentive to participate. The target population for this study included cattle producers in Colorado. The frame used for the stratified random sample was derived via databases from three sources: Colorado Beef Quality Assurance (BQA) Program (n = 541); Colorado Cattlemen’s Association (CCA, n = 1,365); and Colorado Livestock Association (CLA, n = 158). A census of each list (i.e., every eligible name on each list was included in the sample) was used, and duplicates (i.e., individual names that appeared on lists from more than one organization) were removed, resulting in a total sample size of 2,064. Since no publicly available list of cattle producers in Colorado was available, this social survey approach enabled perceptions and attitudes to be evaluated across multiple groups within Colorado’s cattle industry.

Of the 2,064 names and addresses compiled, 1,829 had addresses that met United States Postal Services delivery standards. Thus, each of the 1,829 individuals was assigned a random number through the Microsoft Excel (Redmond, WA) random number generator. This number was added to the respective physical survey to track individually returned surveys. One staff member in Colorado State University’s Mail Production Services Department confidentially oversaw the distribution and tracking of surveys and follow-up mailings. The initial survey, a cover letter, and a postage-paid return envelope were mailed to the address on file for each recipient on May 11, 2021. For participants who did not return a survey, a reminder postcard was sent on June 4, 2021. Finally, non-responders were sent another survey on June 19, 2021. Survey responses were accepted until August 19, 2021.

### Survey Development and Content

A group of researchers with expertise in beef production and the BQA program developed questions for the survey. The survey (available as Supplementary Material) included 40 questions related to: demographics, BQA program knowledge and participation, BQA program perspectives, adoption of BQA principles, and perspectives of value derived from the BQA program. The survey included multiple choice, Likert scale, and free response questions. A group of experts in the survey focal areas reviewed the survey for relevancy and clarity, and feedback was incorporated into the final version.

### Analysis

A total of 546 respondents completed and returned the survey. Sixty-seven households were deemed ineligible because they no longer owned or managed a cattle operation, 10 surveys were duplicate responses (i.e., two surveys were returned from the same address), the identification number was removed on two surveys, and one respondent indicated they were younger than 18 yr old. The final analysis included 466 surveys. A single researcher entered all responses from received surveys into Qualtrics (Provo, UT). If the respondent failed to select an option for a given question, the answer was coded as a missing data point. This data was analyzed in R software (Vienna, Austria). Responses were categorized based on whether the cattle producer was BQA-certified (currently or ever before) or never BQA-certified. Likert scale responses were summarized visually using the Likert package (v1.3.5). Associations between BQA certification status and responses to survey questions were assessed using chi-square tests for independence, with statistical significance determined at P ≤  0.05 level. To examine whether demographic factors, in addition to BQA certification, explained variation in survey responses, we fit ordinal logistic regression models in which Likert responses were collapsed into “Agree,” “Neutral,” “Disagree.” This analysis was performed for questions related to knowledge and adherence to BQA guidelines. BQA certification status, gender, industry segment, and age category were included as predictors. Due to limited number of responses from some industry segments, the models were limited to respondents who were in commercial cow/calf operations, background/stocker/seedstock operations, and feedlots. Odds ratios greater than 1 indicate a higher likelihood of agreeing with the question statement; statistical significance or model predictors was evaluated using a likelihood ratio test.

## RESULTS

### Demographics

There were 466 total respondents to the survey, equaling a 25.5% response rate. Demographics are shown in **Table 1**. A majority of respondents were either “owner and manager/herdsman” (n = 199, 43.5%) or “owner” (n = 219, 47.9%) of their cattle operations. Most respondents had been in the cattle industry for at least 26 yr, and a substantial portion had spent 50 or more years in the cattle industry. Of the respondents, 73% (n = 325) were male, 24% (n = 108) were female, 1% (n = 6), and 2% (n = 7) were non-binary or preferred not to answer, respectively. All regions of Colorado were represented evenly. Moreover, nearly three-quarters of respondents (n = 310, 72.6%) were commercial cow-calf operators, with most respondents having less than 500 head of mature cows.

**Table 1.** Demographics of survey respondents (n = 466)

Variable	*n*	Frequency, %
**Respondent Demographics**		
*Primary Role*		
Contract labor	3	0.7
Hired labor	2	0.4
Manager/herdsman	30	6.6
Owner and manager/herdsman	199	43.5
Owner	219	47.9
Other	4	0.9
*Length of time working in cattle industry*		
Less than 1 yr	5	1.1
1 to 3 yr	5	1.1
4 to 10 yr	32	7.0
11 to 25 yr	80	17.6
26 to 50 yr	189	41.6
More than 50 yr	143	31.5
*Gender*		
Man	325	72.9
Woman	108	24.2
Non-binary	6	1.3
Prefer not to answer	7	1.6
**Operation Characteristics**		
*Region in Colorado*		
Central	74	17.2
Front Range	64	14.8
Northeast	94	21.8
Northwest	63	14.6
Southeast	47	10.9
Southwest	89	20.6
*Industry Segment*		
Backgrounder/preconditioner/grower	17	4.0
Commercial cow/calf	310	72.6
Dairy	7	1.6
Feedyard	28	6.6
Seedstock	43	10.1
Stocker/yearling	17	4.0
*Size, number of mature cows* ^1^		
None	27	6.1
1 to 50	109	24.7
50 to 99	61	13.8
100 to 199	94	21.3
200 to 499	92	20.8
500 to 999	43	9.7
1,000 to 4,999	15	3.4
Greater than 5,000	1	0.2
*Size, number of weaned calves and/or feeder cattle* ^2^		
None	56	12.8
1 to 100	187	42.9
100 to 999	142	32.6
1,000 to 4,999	32	7.3
5,000 to 24,999	10	2.3
25,000 to 49,999	3	0.7
Greater than 50,000	6	1.4

^1^For cow/calf and dairy operations.

^2^For yearling, stocker, backgrounder, and feedlot operations.

### BQA Program Certification and Participation

**Table 2** provides information about BQA program certification and participation. Nearly 95% of respondents (n = 439) had heard of the BQA program, and 72% (n = 313) had participated in BQA training. Fifty-eight percent (n = 272) of respondents reported that they have at one point been BQA-certified. Of the respondents who had been BQA-certified, 69% (n = 193) had done so within the previous 3 yr. Respondents indicated that they received BQA training most often through online training (n = 113, 27.8%) or events hosted by cattle producer associations (n = 96, 23.6%). Over one-third of the respondents (n = 182; 39.1%) reported participating in other cattle or livestock assurance training, certification, or education programs, such as the BQA Transportation Program and the Colorado 4-H Meat Quality Assurance Program.

**Table 2.** Summary of Beef Quality Assurance (BQA) and other quality assurance training related information of survey respondents

Variable	n	Frequency, %
*Heard of BQA (n = 463)*		
Yes	439	94.8
No	24	5.2
*Ever participated in a BQA training (n = 435)*		
Yes	313	72.0
No	120	27.6
I do not know	2	0.5
*Ever been BQA certified (n = 466)*		
Yes	272	58.4
No	194	41.6
*Been BQA certified in last 3* yr *(n = 279)*		
Yes	193	69.2
No	73	26.2
I do not know	13	4.7
*BQA training type (n = 406)*		
Hosted by a cattle producer association	96	23.6
Hosted by a livestock auction market	14	3.4
Hosted by a local livestock Extension agent	61	15.0
Hosted by a private operation	20	4.9
Hosted by a veterinary clinic	18	4.4
Hosted in conjunction with another industry event	18	4.4
Online training	113	27.8
Stockmanship and Stewardship event	41	10.1
Other	25	6.2
*Participation in other cattle or livestock assurance training, certification, and/or education programs (n = 182)*		
BQA Transportation Program	55	30.2
Colorado 4-H Meat Quality Assurance Program	134	73.6
National Dairy FARM^1^ Program	8	4.4
Veal Quality Assurance Program	1	0.5
Youth for the Quality Care of Animals	14	7.7
Other	15	8.2

^1^F.A.R.M. = Farmers Assuring Responsible Management.

### BQA Certification Impacts on BQA Practices

**Figure 1.**
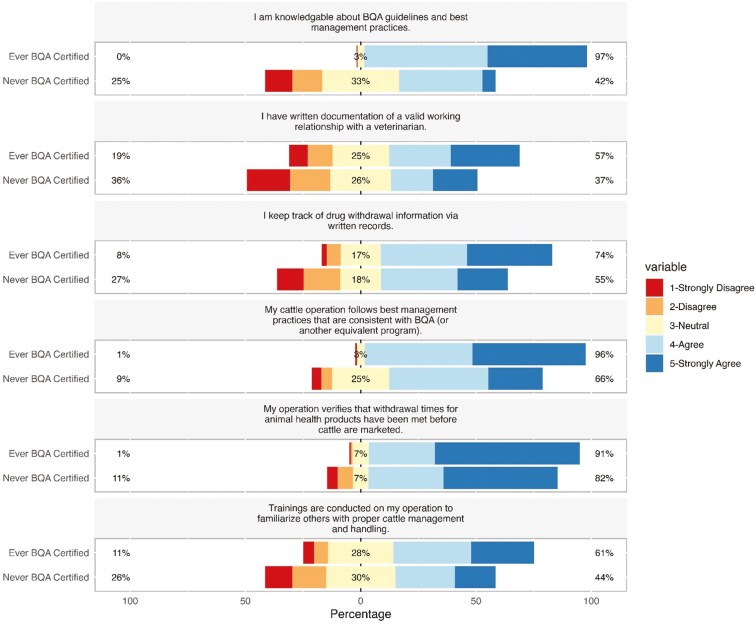
Results of Likert-style questions regarding perceptions of knowledge of and adherence to BQA guidelines. All questions had a significant relationship (P ≤ 0.05) between response and certification status.

There was a significant relationship between BQA certification status and perceived knowledge about and adherence to BQA guidelines, as well as between certification status and an indication of having written documentation of a valid working relationship with a veterinarian (χ^2^ = 171.82 and 20.246, respectively; P < 0.0001; **Figure 1**). When asked about their agreement with the statement “I am knowledgeable about BQA guidelines and best management practices,” 97% (n = 260) of respondents who had ever been BQA-certified agreed or strongly agreed, and 42% (n = 74) of respondents that had never been BQA-certified agreed or strongly agreed. There was also a significant relationship between BQA certification status and agreement with statements regarding proper drug withdrawal protocols and recordkeeping on their operation (χ^2^ = 20.246; P < 0.0001; **Figure 1**). Approximately three-quarters (n = 197; 74%) of ever-BQA-certified respondents agreed or strongly agreed that they keep records of drug usage and withdrawal timelines via written records and 91% (n = 246) agreed or strongly agreed that they verify that withdrawal times are met prior to marketing cattle. Approximately half (n = 96; 55%) of never-certified respondents agreed to keeping written records of drug usage and 82% (n = 146) agreed or strongly agreed that they verify withdrawal times prior to marketing cattle (**Figure 1**). A significant relationship was observed between BQA certification status and agreement with statements about conducting training on their operations to teach proper animal handling techniques to their employees. There was 61% agreement (strongly agree and agree; n = 159) with the statement “There is training conducted to familiarize others with proper cattle management and handling techniques” among those that had ever been BQA-certified, and 44% agreement (strongly agree and agree; n = 74) among those that have never been certified (**Figure 1**).

Additionally, a relationship was observed between BQA certification status and an indication of knowledge of better animal handling and well-being practices (χ^2^ = 171.82, *P* < 0.0001). For example, there was a relationship between certification status and preference for subcutaneous injections over intramuscular injections (χ^2^ = 5.116, *P* = 0.024). A greater percentage of ever-certified respondents indicated a preference for subcutaneous injections over intramuscular injections compared to never-certified respondents (93%, versus 85%; **Table 3**). There was also a relationship between being BQA-certified and preferred injection location used on cattle (χ^2^ = 5.239, *P* = 0.022), with respondents that have ever been BQA-certified more commonly citing “in front of the shoulder” compared with those that have never been BQA-certified (95% versus 88%; **Table 3**), as well as a relationship between BQA certification status and indication of having a written biosecurity protocol on their operation (χ^2^ = 14.254, *P* = 0.0002). Additionally, there was a relationship between certification status and reporting having protocols consistent with guidelines set forth by the BQA Transportation program (χ^2^ = 23.08, *P* < 0.0001).

**Table 3.** Respondent preferences toward and use of cattle management practices

	Ever BQA Certified		Never BQA Certified	
Variable	*n*	Frequency, %	*n*	Frequency, %
*Preferred Injection Type*				
Intramuscular	15	5.7	21	11.9
Subcutaneous	249	93.9	150	84.7
Don’t Know	1	0.4	6	3.4
*Preferred Injection Site*				
In Front of Shoulder (Dewlap)	7	2.7	6	3.4
In Front of Shoulder (Neck)	246	94.6	155	88.1
Lower Rear Leg	0	0.0	1	0.6
Next to Tailhead	0	0.0	1	0.6
Top of Hip	1	0.4	1	0.6
Underneath Front Leg	6	2.3	5	2.8
Other	0	0.0	3	1.7
Don’t Know	0	0.0	4	2.3
*Presence of Formal Biosecurity Plan*				
Yes	49	18.3	11	5.8
No	213	79.5	175	91.6
Don’t Know	6	2.2	5	2.6
*Protocols Consistent with BQAT*				
Yes	160	60.4	73	39.7
No	84	31.7	75	40.8
Don’t Know	21	7.9	36	19.6

### Demographic Factor Impacts on BQA Practices

Ordinal logistic regressions were used to identify whether demographic factors explained variation in survey responses. This analysis was applied to responses to statements presented in Figure 1. There was no relationship (P > 0.05) between any of the additionally tested demographic factors (e.g., gender, age, and industry sector) and responses to the following statements: “I am knowledgeable about BQA guidelines and best management practices,” “I keep track of drug withdrawal information via written records,” and “My cattle operation follows best management practices that are consistent with BQA (or another equivalent program).” It should be noted that, as previously described, BQA certification was associated with responses to these statements (P > 0.05). The results for these analyses are available in supplementary material (Supplemental Tables 1–3). Industry sector (P = 0.0001) and age (P = 0.03) were associated with responses to the following statement: “I have written documentation of a valid working relationship with a veterinarian” (Table 4). Compared to the Cow-Calf sector, both the Feedyard and Stocker/Background/Seedstock sector were associated with greater likelihood of agreement with this statement (OR: 8.65, CI: 2.88, 37.41, and OR: 1.33, CI: 0.78, 2.29, respectively). Compared to respondents under the age of 40 yr, ages 50 to 59 (OR: 0.95, CI: 0.47, 1.90), 60 to 69 (OR: 0.56, CI: 0.28, 1.10), and > 70 yr (OR: 0.69, CI: 0.34, 1.37) were associated with decreased likelihood of agreement with the statement; individuals between 40 and 49 yr had an increased likelihood (OR: 1.69, CI: 0.76, 3.79) of agreeing with the statement as compared with individuals under 40 yr of age. Additionally, gender (P = 0.04) and age (P = 0.02) were associated with responses to the following statement: “My operation verifies that withdrawal times for animal health products have been met before cattle are marketed” (Table 5). Compared to men, both women (OR: 3.50, CI: 1.28, 12.37) and non-binary individuals or respondents that preferred not to answer (OR: 1.45, CI: 0.23, 28.41) had an increased likelihood of agreeing with the statement. All age categories had a decreased likelihood of agreeing with that statement as compared to individuals under the age of 40 (40 to 49 yr, OR: 0.37, CI: 0.05, 20.04; 50 to 59 yr, OR: 0.24, CI: 0.04, 0.98; 60 to 69 yr, OR: 0.77, CI: 0.11, 3.55; > 70 yr, OR: 0.21, CI: 0.03, 0.78). Lastly, industry sector (P = 0.01) and age (P = 0.01) were associated with responses to the following statement: “Trainings are conducted on my operation to familiarize others with proper cattle management and handling” (Table 6). Compared to the Cow-Calf sector, the Feedyard had a greater likelihood of agreeing with that statement (OR: 3.69, CI: 1.41, 11.60) and the Stocker/Background/Seedstock sector had a decreased likelihood of agreeing with that statement (OR: 0.81, CI: 0.48, 1.36). All age categories had a greater likelihood of agreeing with that statement as compared to individuals under the age of 40 (40 to 49 yr, OR: 2.89, CI: 1.34, 6.39; 50 to 59 yr, OR: 2.06, CI: 1.04, 4.10; 60 to 69 yr, OR: 1.85, CI: 0.95, 3.60; > 70 yr, OR: 1.05, CI: 0.54, 2.04).

**Table 4.** Multivariable ordinal logistic regression model for agreement with the statement: I have written documentation of a valid working relationship with a veterinarian. The answers were categorized as Agree, Neutral, and Disagree for this analysis; higher odds ratios indicate greater likelihood of agreeing with this statement

Variable	Estimate	SE	*P*-value	OR (95% CI)
**BQA Certification**			0.002	
No	*Referent*	–	–	–
Yes	0.66	0.22		1.94 (1.27, 2.98)
**Gender**			0.14	
Man	*Referent*	–	–	–
NonBinary and Prefer Not to Answer	1.50	0.83		4.48 (1.01, 31.26)
Woman	0.06	0.25		1.06 (0.64, 1.77)
**Industry Sector**			0.0001	
Cow-calf	*Referent*	–	–	–
Feedyard	2.16	0.65		8.65 (2.88, 37.41)
Stocker/Background/Seedstock	0.29	0.27		1.33 (0.78, 2.29)
**Age**			0.03	
< 40 yr	*Referent*	–	–	–
40 to 49 yr	0.52	0.40		1.69 (0.76, 3.79)
50 to 59 yr	-0.06	0.35		0.95 (0.47, 1.90)
60 to 69 yr	-0.58	0.34		0.56 (0.28, 1.10)
> 70 yr	-0.37	0.34		0.69 (0.34, 1.37)

**Table 5.** Multivariable ordinal logistic regression model for agreement with the statement: My operation verifies that withdrawal times for animal health products have been met before cattle are marketed. The answers were categorized as Agree, Neutral, and Disagree for this analysis; higher odds ratios indicate greater likelihood of agreeing with this statement

Variable	Estimate	SE	*P*-value	OR (95% CI)
BQA Certification			0.16	
No	*Referent*	–	–	–
Yes	0.50	0.36		1.65 (0.82, 3.33)
Gender			0.04	
Man	*Referent*	–	–	–
NonBinary and Prefer Not to Answer	0.37	1.06		1.45 (0.23, 28.41)
Woman	1.25	0.57		3.50 (1.28, 12.37)
Industry Sector			0.07	
Cow-calf	*Referent*	–	–	–
Feedyard	1.08	1.08		2.94 (0.55, 54.62)
Stocker/Background/Seedstock	1.07	0.56		2.93 (1.08, 10.26)
Age			0.02	
< 40 yr	*Referent*	–	–	–
40 to 49 yr	-1.00	0.90		0.37 (0.05, 2.04)
50 to 59 yr	-1.43	0.80		0.24 (0.04, 0.98)
60 to 69 yr	-0.27	0.84		0.77 (0.11, 3.55)
> 70 yr	-1.60	0.78		0.21 (0.03, 0.78)

**Table 6.** Multivariable ordinal logistic regression model for agreement with the statement: Trainings are conducted on my operation to familiarize others with proper cattle management and handling. The answers were categorized as Agree, Neutral, and Disagree for this analysis; higher odds ratios indicate greater likelihood of agreeing with this statement

Variable	Estimate	SE	*P*-value	OR (95% CI)
BQA Certification			< 0.001	
No	*Referent*	–	–	–
Yes	0.75	0.22		2.11 (1.38, 3.24)
Gender			0.45	
Man	*Referent*	–	–	–
NonBinary and Prefer Not to Answer	0.90	0.75		2.47 (0.62, 12.43)
Woman	0.002	0.25		1.00 (0.61, 1.65)
Industry Sector			0.01	
Cow-calf	*Referent*	–	–	–
Feedyard	1.31	0.53		3.69 (1.41, 11.60)
Stocker/Background/Seedstock	-0.21	0.26		0.81 (0.48, 1.36)
Age			0.01	
< 40 yr	*Referent*	–	–	–
40 to 49 yr	1.06	0.39		2.89 (1.34, 6.39)
50 to 59 yr	0.72	0.34		2.06 (1.04, 4.10)
60 to 69 yr	0.61	0.33		1.85 (0.95, 3.60)
> 70 yr	0.05	0.33		1.05 (0.54, 2.04)

### BQA Certification Impacts on Cattle Marketing

**Table 7** provides information about the proportions of both ever-BQA-certified and never-BQA-certified respondents who promoted various claims when marketing their cattle. There was a relationship between BQA certification and using BQA certification as a marketing strategy (χ^2^ = 93.001, *P* < 0.0001). However, there was not a significant relationship between being BQA-certified and using marketing claims such as natural, non-hormone treated, and organic cattle (χ^2^ = 1.13, *P* = 0.288; χ^2^ = 0.036, *P = *0.851; χ^2^ = 1.733, *P* = 0.188, respectively). Similarly, there was no evidence of a relationship between certification status and marketing cattle with vaccines or animal health protocol characteristics (*P* = 1.00).

**Table 7.** Respondent cattle marketing strategies (n = 466)

	Ever BQA Certified		Ever BQA Certified Never BQA Certified	
Variable	*n*	Frequency, %	*n*	Frequency, %
**Marketing Claims**				
*Promoted BQA Certification*				
Yes	120	76.9	5	2.9
No	136	23.1	165	97.1
*Promoted Natural*				
Yes	117	45.7	68	40.0
No	139	54.3	102	60.0
*Promoted Non-Hormone Treated*				
Yes	106	41.4	68	40.0
No	150	58.6	102	60.0
*Promoted Organic*				
Yes	4	1.6	7	4.1
No	252	98.4	163	95.9
*Promoted Vaccinations/Animal Health Products*				
Yes	188	73.4	125	73.5
No	68	26.6	45	26.5
*Promoted Weaning/Preconditioning Status*				
Yes	180	70.3	114	67.1
No	76	29.7	56	32.9
*Promoted Other Claims*				
Yes	19	7.4	17	10.0
No	237	92.6	153	90.0
*Asked for proof of BQA certification at time of sale*				
Yes	50	19.2	5	2.7
No	210	80.8	177	97.3
*Observed premiums for BQA certification*				
Yes	24	10.6	8	5.8
No	202	89.4	131	94.2

**Table 7** also shows the proportions of ever- and never-BQA-certified respondents who indicated needing to show proof of certification status or receiving a premium for being certified at the time of sale. There was an association observed between certification status and being required to show evidence of BQA certification at the time of sale (χ^2^ = 26.705, *P* < 0.0001). However, there was no evidence of a relationship between BQA certification status and an indication of noticing a premium on cattle from BQA-certified operations (χ^2^ = 2.965, *P* = 0.227).

### BQA Certification Perceptions

**Figure 2.**
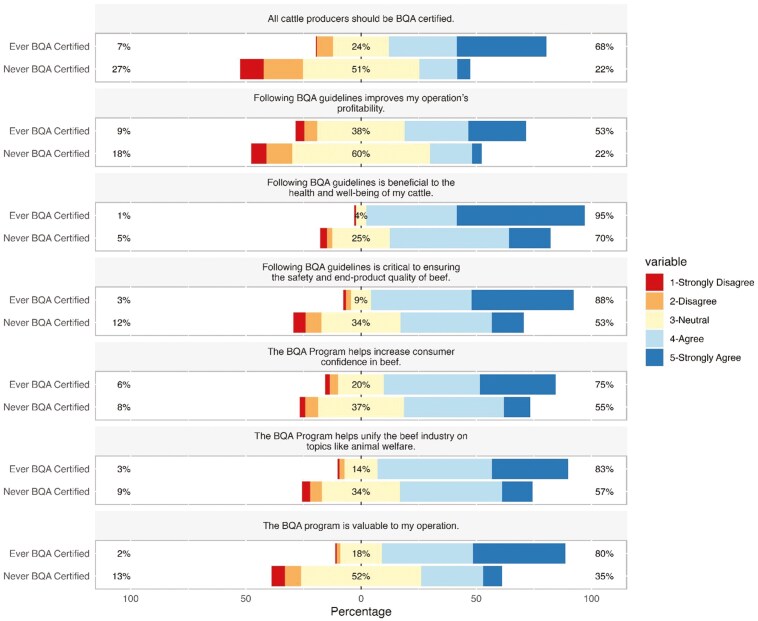
Results of Likert-style questions regarding perceived value of BQA programs. All questions had a significant relationship (P ≤ 0.05) between response and certification status.

The analysis uncovered a relationship between BQA certification status and the belief that BQA certification increases consumer confidence in beef production (χ^2^ = 18.886, *P* < 0.0001). For example, 75% (n = 204) of certified respondents (now or ever before) and 55% (n = 107) of never-certified respondents agreed or strongly agreed with the statement “BQA certification increases consumer confidence in beef production” (**Figure 2**). Similarly, there was a significant relationship between being ever-BQA-certified and believing that BQA increases beef industry unity (χ^2^ = 33.924, *P* < 0.0001), as shown by the 83% (n = 226) of ever-BQA-certified respondents that agreed or strongly agreed with the following statement: “The BQA program helps to unify the beef industry on topics like animal welfare,” compared with only 57% (n = 111) of the never-certified group that agreed or strongly agreed with that statement (**Figure 2**). Additionally, there was a relationship between BQA certification status and responses to the statement “All cattle producers should be BQA-certified” (χ^2^ = 93.632, *P* < 0.0001). Similarly, there was a relationship between BQA certification and agreeing or strongly agreeing that “BQA certification was critical to ensuring safety and end-product quality of beef” (χ^2^ = 65.291, *P* < 0.0001; **Figure 2**) as well as agreeing or strongly agreeing that “Following BQA guidelines is beneficial to their herd’s health and wellbeing” (χ^2^ = 51.19, *P* < 0.0001). A relationship was observed between being ever-BQA-certified and believing that BQA certification was valuable (χ^2^ = 89.953, *P* < 0.0001). Eighty percent of respondents who have ever been certified agreed that being BQA-certified was valuable to their operations, whereas 52% (n = 101) of never-certified respondents were neutral, and 35% (n = 68) agreed or strongly agreed (**Figure 2**). Lastly, there was a relationship between BQA certification and agreement with the statement “BQA certification increases profitability on their operations (χ^2^ = 39.133, *P* < 0.0001). In the ever-BQA-certified group, 53% (n = 144) agreed or strongly agreed that BQA certification brings more profit, while 24% (n = 65) were neutral, and only 7% (n = 19) disagreed (**Figure 2**). For the same question, only 22% (n = 43) of never-certified respondents agreed or strongly agreed, 51% (n = 99) were neutral, and 27% (n = 52) disagreed or strongly disagreed (**Figure 2**).

## DISCUSSION

Although the BQA program has existed for several decades, there are not widely available reports of beef producers’ perceptions of the program. To understand how producers perceive the program to thus inform state programming, a survey was created and sent to Colorado beef producers. Beef Quality Assurance is a free, voluntary program offered by NCBA and implemented by each state, aimed to provide beef producers with best management practices that improve the safety and quality of beef and promote cattle well-being. Colorado has over 12,000 beef cattle producers with over 2.6 million head of cattle, including beef and dairy cattle, calves, cattle on feed, and all other cattle classes (USDA NASS, 2022a). The majority of respondents in the current survey represented the cow-calf sector and had less than 500 head of cattle, similar to national statistics (USDA NASS, 2022b). The current survey reached approximately 15% of Colorado beef producers and collected responses from both ever-BQA-certified and never-BQA-certified respondents. In the current survey, approximately 73% of respondents identified as men, in line with national statistics reporting that the majority of farmers are men (USDA NASS, 2022c). Similar to United States statistics of beef producers, where the average age is over 50 yr (USDA NASS, 2022c), most respondents in the current survey were between 35 and 64 yr old.

### BQA Program Certification and Participation

Nationally, the BQA program has over 210,000 BQA-certified producers, representing 27% of the cattle producers (personal communication, J. Fitz-Brewer). In the current survey, 95% of respondents indicated having heard of the BQA program, 72% had indicated participating in at least one BQA training, and 58% indicated having, at some point, currently or formerly, held a BQA certification. Although nearly all respondents had heard of the program, only about half had been certified. This could be attributed to many factors, including, but not limited to, the accessibility and communication of training opportunities and the producers’ perceived value of holding the certification.

Two training certification modalities are offered to help producers become BQA-certified in Colorado: (1) in-person training workshops and (2) the more recently launched, self-paced online modules, both requiring recertification every 3 yr. The online training modules have become more popular with CO beef producers, as reflected in the current study and the CO BQA program statistics (NCBA, 2020). Advantages and preferences of producers for the online training modules include 24/7 availability, widespread broadband access, course offerings in English and Spanish, and self-paced modules (NCBA, 2020; Baumann et al., 2023). In a survey of behavioral health providers seeking workplace training in various modalities, Mallonee et al. (2018) reported that completing work-related training online led to an equal or increased satisfaction rate in the overall training experience compared with in-person modalities. Although the current study did not ask producers why they selected the different training modalities, the convenience coupled with an equal or increased satisfaction rate of online training modalities could influence producers to become BQA-certified and/or recertified. Comparatively, the in-person BQA certifications are led by the state BQA coordinator, veterinarian, or extension agent trained by the state BQA coordinator. Attending in-person workshops and training events can require additional time (e.g., travel, attendance time, etc.) and thus requires more coordination on behalf of both the trainers and cattle producers. Despite the challenges, in-person training may increase motivation to learn and increase the application of knowledge following the workshop compared to those who received the information via an online training module or program (Dimeff et al. 2015). In the current study, numerous respondents attended in-person trainings, which provided the benefit of completing BQA within a structured timeline and allowed for interaction between the trainers and cattle producers. There are advantages to both training modalities offered, and providing both options allows the BQA program to reach more cattle producers and provide them with various modes to engage and learn.

### Knowledge of and Adherence to BQA Practices

In the current survey, most respondents felt they were knowledgeable about BQA guidelines and best management practices; almost all of the ever-BQA-certified respondents felt they were knowledgeable. One interesting result was that 66% of the never-BQA-certified respondents reported following “best management practices that are consistent with BQA.” However, only 44% of the never-certified respondents reported that they were knowledgeable about BQA guidelines and best management practices. Perhaps a portion of the never-BQA-certified group participates in other animal care and verification programs (e.g., Progressive Beef, Global Animal Partnership, Certified Humane), which share many of the same foundational best management practices for cattle care.

Some specific components of animal care related to herd health and safety were asked in the survey. In the current study, a greater proportion of ever-certified respondents agreed that their operations follow best management practices consistent with BQA guidelines than those that have never been certified. For example, when asked questions about respondents’ preferences for subcutaneous or intramuscular injections and preferred injection sites, a greater proportion of respondents who had ever been certified responded with preferences aligned with BQA guidelines, compared with those who had never been certified. Although there was still a lower proportion overall, the proportion of never-BQA-certified respondents whose preferences aligned with BQA guidelines was still above 80%. This finding aligns with national survey data, which reported that most surveyed feedyards indicated that the location used to administer injectable products (e.g., in the neck, shoulder, side, or leg) was somewhat or very important (USDA APHIS, 2012). This is especially crucial because improper injections can lead to injection-site lesions, which can, in turn, negatively impact animal welfare, meat quality, and producer income (Cresswell and Remnant, 2017). Another example of adherence to herd health and safety best practices asked in the current survey was regarding verifying drug withdrawal times being met prior to marketing cattle. It was determined that both women and non-binary and individuals who preferred not to indicate their gender were more likely to agree that they followed this practice, when compared to men, and that respondents in age categories older than 40 were less likely to agree to following this practice, when compared to those under the age of 40. Although not specific to animal care programs, prior studies have identified impacts of gender on rule abidance, in this case following withdrawal times, with women often demonstrating higher rule abidance than men (DeHart-Davis, 2009; Portillo and DeHart-Davis, 2009). Additionally, these results align with other studies that have found older livestock producers to be behind the curve on the adoption of best practices (Johnson et al., 2010).

In addition to asking about specific health practices, the current survey asked about the documentation of a veterinarian-client-patient relationship (VCPR). These results indicate that over half of ever-certified respondents agreed with having written documentation of a relationship with a veterinarian, compared to only 37% of those who had never been certified. A strong and reliable VCPR is an essential component of a herd health program. A report put forth by the American Association of Bovine Practitioners regarding key VCPR principles states that it is crucial to have and utilize a VCPR on feedlot operations to ensure proper prevention and treatment protocols, legal and ethical dispensing of drugs, and consistent oversight and training of employees (AABP, 2020). Having a documented relationship with a veterinarian can improve the use of animal health products, adherence to withdrawal times, the creation and implementation of effective biosecurity protocols, and the training of all employees on these practices (Smith et al., 2015). Further, the current study found that age and industry sector were significantly associated with respondents’ likelihood of agreeing that they have documentation of a VCPR on their operation. The effect of industry sector on this statement could be related to the difference in the frequency of audits taking place on feedyards as compared with other supply chain sectors. It is recommended by BQA that feedyards are audited annually, and one key component of this audit is documentation of a VCPR (BQA, 2021). Additionally, feedyards tend to have more verified programs, such as Certified Humane, that require documentation of a VCPR.

Overall, our data suggests an association between BQA certification and adherence to best practices in cattle production, so attention should still be paid to educating and training producers. Our finding that those respondents who have ever been BQA-certified more frequently followed BQA guidelines was not surprising, and earlier studies have shown the effectiveness of cattle producer education on improving herd health, animal well-being, and even profitability (Adams et al., 2016; Klopatek et al., 2022). Often, these trainings are conducted by the veterinarian associated with the operation, allowing employees to serve as additional technicians who can perform health tasks that don’t require a veterinarian to be present (i.e., overall health checks, obstetrics, specific drug administration techniques). This could lead to a quicker reaction time in some instances of morbidity, as well as to an increase in employees’ knowledge base to be utilized when creating preventative care plans and biosecurity protocols, leading to improved herd health. These employees can also be trained in quality assurance techniques, whether those put out by BQA or by other similar organizations. A study by Ceballos et al. (2018) found that training employees on proper cattle management and handling techniques leads to better attitudes and behavior toward cattle, promoting a more positive animal-human interaction and increasing animal welfare. Nearly half of current study respondents agreed that they hold trainings on their operations to familiarize others with proper cattle management and handling; respondent age and industry sector were associated with the likelihood of agreeing with this. The likelihood of agreeing increased when comparing the Cow-Calf sector when comparing to both to the Stocker/Backgrounder/Seedstock sector the Feedyard sector. This could be because, as one gets closer to the slaughter facilities in terms of production cycles, they become more visible to the public and thus get more attention surrounding meat quality, food safety, and animal welfare concerns, which can often be associated with more verification and/or assessments of programs. The likelihood of agreement with the statement regarding conducting trainings on one’s operation also increased when comparing respondents over 40 to those under the age of 40. As stated previously, the effect of age on a person indicating they follow best practices can vary between individuals. Again, having knowledge of the benefits of holding trainings can lead to producers being more likely to do so, but there are numerous other factors, such as pressure from consumers and other industry members, that ultimately lead to the final decision to start and uphold the regular training schedule.

### The Value of BQA Certification

The primary goal of BQA certification is to promote best management practices that ensure animal well-being, food safety, and quality, while fostering consumer confidence that beef is raised safely and humanely. The current study results demonstrate that a large proportion of ever-BQA-certified individuals, and many never-certified individuals, agree that the program has these benefits. The current research found that currently or formerly BQA-certified respondents saw value in being certified in terms of increased profitability, decreased losses due to animal health and welfare issues, and a resulting increase in consumer confidence in the beef produced. Regarding the economic value of being BQA-certified, the data showed that ever-BQA-certified respondents were more likely to use claims of their BQA certification in their marketing strategy. There is some evidence that calves raised by producers with BQA certification could increase the selling price of beef calves marketed through online video auction systems. A study conducted by Mooney et al. (2019) found an average premium of $2.69/cwt for lots of cattle being sold in an online video auction that mentioned BQA, compared to those lots that did not have a BQA mention. In the current study, 53% of ever-BQA-certified respondents reported increased ranch profitability due to following BQA guidelines. Previous research on the economics of on-farm animal welfare programs found no significant change in the profitability of cattle producers using quality assurance schemes for marketing beef (Tsakiridis et al., 2021). This suggests that BQA certification does not necessarily lead to direct revenue increases through higher cattle prices at the time of sale.

DeCoite (2021) found in a study that the two most common reasons producers consider BQA certification are to receive higher premiums for their cattle and to recoup the costs associated with producer verification when selling cattle through programs, yet current results did not show a strong association between being BQA certified and obtaining a premium at the time of sale. A study conducted by Gross et al. (2021) suggests that the economic value derived from quality assurance programs could vary by how the program is described to the consumer and could explain the low level of premiums observed from cattle originating from BQA-certified ranches. For example, Gross et al. (2021) found that consumers' willingness to pay (WTP) for organic pork products was greater than for pork produced under specified welfare standards and that there were greater numerical increases in WTP for broad welfare standards compared with specific standards. This could be due, in part, to a lack of brand recognition of BQA at the meat case in retail stores. Without an increase in consumer WTP at retail stores, packers and feedyards are less likely to offer producers a premium for their cattle raised under BQA guidelines.

In addition to a lack of brand recognition, most consumers find that any practice that negatively impacts animal welfare is unacceptable and should not be allowed in the industry (Alonso et al., 2020); therefore, it is more of an expectation that producers follow best management practices and proper handling techniques, i.e., following best management practices like BQA are an expectation, not an added bonus to a program. Thus, consumers are not likely to increase WTP simply for producers following their baseline expectations surrounding animal handling. Despite these findings from other researchers, animal well-being has received greater consumer attention in the recent past (Alonso et al., 2020; FMI, 2022), and the renewed consumer interest in further improving animal well-being during production and slaughter could be an opportunity for beef producers (NCBA Consumer Insights, 2022).

Regardless of whether premiums are due to BQA certification, it is important to note that financial benefits of animal welfare practices exist outside of the selling price of calves, including reduced mortality, improved health, reduced disease prevalence, improved meat quality, and increased job satisfaction (Dawkins, 2016). These findings express the same sentiment as shared in the current survey, where 95% of producer respondents who are or have ever been BQA-certified agreed that BQA practices increased herd health, and 80% agreed that being BQA-certified was valuable to their operations.

Improved animal well-being plays a pivotal role in maintaining consumer trust in and demand for beef and may not have a quantifiable value from a marketing (labeling) perspective. Ultimately, producing high quality and safe beef that is desired by consumers is the aim of BQA, and the current results indicate that Colorado beef producers are working to achieve that aim. According to a survey conducted by NCBA, animal welfare is the top concern for American consumers when purchasing beef when compared with other concerns such as sustainability and price (NCBA Consumer Insights, 2022). In that consumer survey when asked if any concerns are present regarding how food animals are raised, more than half of the respondents said yes, and of those respondents, the most selected issue was animal welfare. Additionally. in a survey of 1,000 meat consumers conducted by Spain et al. (2018), 78% of respondents believed there should be a third-party auditor on ranches to ensure animal welfare, and a majority of respondents indicated a willingness to pay for meat raised under proper welfare standards, both in retail stores and restaurants. Further, Thibault et al. (2022) reported that 83% of respondents in a survey of 1,000 U.S. consumers indicated they would likely switch to a meat, egg, or dairy brand that guaranteed animals would be raised with higher animal welfare standards. These findings by previous authors suggest that improving animal welfare baseline expectations will influence consumers purchasing behavior. Implementing BQA best practices on producers’ operations is one method the United States beef industry has and can continue to employ to meet the consumer demand for higher welfare standards while improving herd well-being, a win-win situation for consumers and producers alike.

## CONCLUSIONS

This study found that Colorado cattle producers perceived both monetary and intangible benefits from BQA certification. Our data indicate that many producers adhere to BQA guidelines, and BQA certification was linked to following best management practices in cattle raising. Both current and former BQA-certified producers believed their cattle resulted in higher beef quality, improved safety, and enhanced animal welfare, better meeting evolving consumer expectations around those topics. Even respondents who were never BQA-certified acknowledged the program’s value in enhancing animal well-being and meat quality, ultimately improving consumer confidence in the beef industry.

## Supplementary Material

txaf057_suppl_Supplementary_Tables_S1-S31

txaf057_suppl_Supplementary_Material
