# Supplementary material for: Survey of Colorado beef producers’ perceptions of the Beef Quality Assurance program
Source: Transl Anim Sci. 2025 Apr 29;9:txaf057. doi: 10.1093/tas/txaf057 (PMC12357238; doi:10.1093/tas/txaf057)
Supplement: txaf057_suppl_Supplementary_Tables_S1-S31 [file txaf057_suppl_supplementary_tables_s1-s31.pdf]

Colorado State University: Supplemental tables associated with TAS paper “Survey of Colorado beef producers’ perceptions of Beef Quality Assurance.”

Supplemental Table 1. Multivariable ordinal logistic regression model for agreement with the statement: I am knowledgeable about BQA guidelines and best management practices. The answers were categorized as Agree, Neutral, and Disagree for this analysis; higher odds ratios indicate greater likelihood of agreeing with this statement.

**Variable**	**Estimate**	**SE**	***P*-value**	**OR (95% CI)**
**BQA Certification**			< 0.0001	
No	*Referent*	–	–	–
Yes	3.94	0.44		51.39 (23.27, 132.62)
**Gender**			0.94	
Man	*Referent*	–	–	–
NonBinary[1]	-0.23	0.89		0.80 (0.18, 3.75)
Woman	0.06	0.38		1.06 (0.50, 2.29)
**Industry Sector**			0.49	
Cow-calf	*Referent*	–	–	–
Feedyard	-0.69	0.75		0.50 (0.13, 2.24)
Stocker/Background/ Seedstock	0.26	0.41		1.30 (0.59, 2.96)
**Age**			0.87	
< 40 years	*Referent*	–	–	–
40 to 49 years	-0.47	0.60		0.63 (0.18, 2.08)
50 to 59 years	0.04	0.57		1.04 (0.33, 3.18)
60 to 69 years	-0.22	0.53		0.80 (0.27, 2.29)
>70 years	-0.22	0.53		0.74 (0.26, 2.01)

[1]This included individuals that responded as NonBinary and Prefer Not to Answer.

Supplemental Table 2. Multivariable ordinal logistic regression model for agreement with the statement: I keep track of drug withdrawal information via written records. The answers were categorized as Agree, Neutral, and Disagree for this analysis; higher odds ratios indicate greater likelihood of agreeing with this statement.

Variable	Estimate	SE	*P*-value	OR (95% CI)
**BQA Certification**			< 0.0001	
No	*Referent*	–	–	–
Yes	1.01	0.24		2.74 (1.73, 4.38)
**Gender**			0.30	
Man	*Referent*	–	–	–
NonBinary[1]	0.34	0.77		1.41 (0.36, 6.96)
Woman	0.43	0.29		1.54 (0.88, 2.78)
**Industry Sector**			0.07	
Cow-calf	*Referent*	–	–	–
Feedyard	1.13	0.66		3.11 (1.01, 13.64)
Stocker/Background/ Seedstock	-0.27	0.29		0.76 (0.44, 1.36)
**Age**			0.052	
< 40 years	*Referent*	–	–	–
40 to 49 years	0.43	0.44		1.53 (0.66, 3.66)
50 to 59 years	0.77	0.42		2.17 (0.98, 4.91)
60 to 69 years	-0.17	0.36		0.84 (0.41, 1.71)
>70 years	-0.13	0.37		0.88 (0.42, 1.79)

[1]This included individuals that responded as NonBinary and Prefer Not to Answer.

Supplemental Table 3. Multivariable ordinal logistic regression model for agreement with the statement: My cattle operation follows best management practices that are consistent with BQA (or another equivalent program). The answers were categorized as Agree, Neutral, and Disagree for this analysis; higher odds ratios indicate greater likelihood of agreeing with this statement.

Variable	Estimate	SE	*P*-value	OR (95% CI)
**BQA Certification**			< 0.0001	
No	*Referent*	–	–	–
Yes	2.38	0.38		10.80 (5.37, 23.61)
**Gender**			0.41	
Man	*Referent*	–	–	–
NonBinary[1]	-0.29	0.88		0.75 (0.14, 5.97)
Woman	0.53	0.43		1.70 (0.76, 4.11)
**Industry Sector**			0.57	
Cow-calf	*Referent*	–	–	–
Feedyard	-0.20	0.73		0.82 (0.23, 3.94)
Stocker/Background/ Seedstock	0.43	0.45		1.53 (0.67, 3.87)
**Age**			0.15	
< 40 years	*Referent*	–	–	–
40 to 49 years	1.79	0.86		6.01 (1.31, 43.41)
50 to 59 years	0.10	0.53		1.10 (0.38, 3.09)
60 to 69 years	0.37	0.52		1.45 (0.51, 3.98)
>70 years	0.34	0.50		1.41 (0.51, 3.76)

[1]This included individuals that responded as NonBinary and Prefer Not to Answer.
