# Supplementary material for: Survey of Colorado beef producers’ perceptions of the Beef Quality Assurance program
Source: Transl Anim Sci. 2025 Apr 29;9:txaf057. doi: 10.1093/tas/txaf057 (PMC12357238; doi:10.1093/tas/txaf057)
Supplement: txaf057_suppl_Supplementary_Material [file txaf057_suppl_supplementary_material.pdf]

Perceptions of The Colorado Beef Quality Assurance (BQA) Program
Producer Survey

Questions:

1. Are you a cattle producer in Colorado?

_____ Yes *Please go to next question*
_____ No *Thank you for your time, you do not need to complete the rest of this survey. Please return it in the stamped envelope.*

2. In which segment of the cattle industry do you **primarily** operate? (mark only one)

_____Backgrounder/preconditioner
_____Beef seedstock
_____Calf ranch (male dairy calves)
_____Commercial cow/calf
_____Dairy
_____Dairy replacement heifer facility
_____Feedyard
_____Stocker/yearling
_____Other (please specify):

3. Other than via this survey, have you ever heard of Beef Quality Assurance (BQA) or the BQA Program?

_____ Yes *Continue to next question*
_____ No *Skip to question 8*

4. Have you ever participated in a BQA training (either on-line or in-person)?

_____Yes *Continue to next question*
_____No *Skip to question 8*
_____I don't know *Continue to next question*

5. Have you ever been BQA Certified?

_____Yes *Continue to next question*
_____No *Skip to question 8*
_____I don't know *Skip to question 8*

6. If you participated in a BQA training, what type of training was it? (mark all that apply)

_____Hosted by a cattle producers' association
_____Hosted by a livestock auction market
_____Hosted by a local livestock Extension agent
_____Hosted by a private operation
_____Hosted in conjunction with another cattle industry event (i.e., Colorado Farm Show, Four States Ag Expo, etc.)
_____On-line training
_____Stockmanship & Stewardship event
_____Other (please specify):

7. Did you complete your BQA Certification within the last 3 years?

_____Yes
_____No
_____I don't know

8. Have you ever participated in any other cattle or livestock assurance training, certification, and/or education programs? (mark all that apply)

_____BQA Transportation (BQAT) Program
_____Colorado 4-H Meat Quality Assurance (MQA) Program
_____National Dairy F.A.R.M. (Farmers Assuring Responsible Management) Program
_____Veal Quality Assurance (VQA) Program
_____Youth for the Quality Care of Animals (YQCA)
_____Other (please specify):

9. Are you aware of the BQA self-assessment resources available to cow/calf, stocker/backgrounder, and feedyard operations to benchmark in areas such as animal welfare, cattle handling, and record keeping?

_____Yes *Continue to next question*
_____No *Skip to question 11*

10. Have you ever completed a BQA self-assessment on your cattle operation?

_____Yes
_____No

_____ I don't know

11. Have you ever been audited by a 3rd party related to your cattle production practices?

_____Yes *Continue to next question*
_____No *Skip to question 14*
_____I don't know *Skip to question 14*

12. If you answered yes to the previous question, what was the program?

13. If you have been 3rd party audited, did it involve verifying your BQA Certification status and/or adherence to BQA guidelines?
_____Yes
_____No
_____I don't know

14. If you have been BQA Certified, what are the reasons you choose to follow best management practices that are consistent with BQA on your operation? (mark all that apply)

_____I have never been BQA Certified
_____I am committed to continuous improvement on my cattle operation
_____I receive a premium when I sell my cattle
_____I want to stay current with industry expectations
_____It's required by the buyer of my cattle
_____It's the right thing to do
_____Other (please specify):

15. If you were BQA Certified at one time, but aren't currently, why is that the case? (mark all that apply)

_____I have never been BQA Certified
_____Buyers are not asking for documentation of my BQA Certification status
_____I don't have time
_____I have limited access to the internet
_____It costs too much money
_____It's not required for me to participate
_____There was no value to me in getting re-certified
_____There is no financial incentive for me to participate

_____The opportunities to get re-certified aren't convenient or readily available
_____Other (please specify):

16. If you have **never** been BQA Certified, what are possible reasons why you are not? (mark all that apply)

_____I have been BQA Certified
_____Buyers are not asking for documentation of my BQA Certification status
_____I don't have time
_____I don't really know what BQA is
_____I don't know how to become certified/access materials
_____I have limited access to the internet
_____It costs too much money
_____It's not required for me to participate
_____There is no financial incentive for me to participate
_____The opportunities to get re-certified aren't convenient or readily available
_____Other (please specify):

17. Would it be helpful to your operation to have BQA training materials provided in Spanish?

_____Yes
_____No
_____I don't know

18. Do you think the Colorado BQA Program could be improved?

_____Yes
_____No
_____I don't know

29. Provide 3 to 5 words for each of the following:

What is one reason you are **supportive** of the CO BQA Program:

What is one **area for improvement** within the CO BQA Program:

19. Please indicate your level of agreement with the following statements:

	Strongly Disagree	Disagree	Neutral	Agree	Strongly Agree
a. I am knowledgeable about B QA guidelines and best management practices	1	2	3	4	5
b. I have written documentation of a valid working relationship with a veterinarian (e.g., veterinary-client-patient-relationship).	1	2	3	4	5
c. I keep track of drug withdrawal information via written records	1	2	3	4	5
d. My cattle operation follows best management practices that are consistent with BQA (or another equivalent program)	1	2	3	4	5
e. My operation verifies that withdrawal times for animal health products (e.g., antibiotics, pain relief, dewormers, vaccines) have been met before cattle are marketed.	1	2	3	4	5
f. Trainings are conducted on my operation to familiarize others (employees, family, friends, etc.) with proper cattle management and handling	1	2	3	4	5

20. If an animal health product can be injected either intramuscularly (IM, into-the-muscle) or subcutaneously (SubQ, under-the-skin), which route do you use **most** often on your operation?

_____Intramuscular (IM)
_____Subcutaneous (SubQ)
_____I don’t know

21. When administering injectable animal health products on your operation, where is your **preferred** site of administration on an animal? (mark only one)

_____Along the topline (on either side of the backbone)
_____In front of the shoulder (dewlap region)
_____In front of the shoulder (neck region)
_____Lower rear leg
_____Next to the tailhead (caudal fold)
_____Top of the hip
_____Underneath the front leg (armpit)
_____I don't know
_____Other (please specify):

22. Do you have a formal (i.e., written) biosecurity plan for disease prevention on your operation?

_____Yes
_____No
_____I don't know

23. Which of the following do you use as your **primary** driving tool when working/sorting cattle? (mark only one)

_____Cane
_____Electric prod (hot shot)
_____Flag
_____Rattle paddle
_____Sorting stick
_____I don't use any driving tools
_____Other (please specify):

24. Do you have a protocol in place that is consistent with proper **BQA Transportation guidelines**, possibly including fitness of animals for transport, loading/unloading, and risk and emergency management?

_____ Yes
_____ No
_____ I don't know

25. When marketing your cattle, do you promote any of the following characteristics? (mark all that apply)

_____BQA Certification status
_____Natural
_____Non-hormone treated cattle (NHTC)
_____Organic
_____Vaccination or animal health protocols
_____Weaning and/or preconditioning status
_____Other (please specify):

26. Have buyers of your cattle ever required evidence of your BQA Certification status?

_____Yes
_____No
_____I don't know

27. Have you ever observed any increase in the selling price of your cattle specifically due to your BQA Certification status?

_____Yes
_____No
_____I don't know

28. If you purchase outside cattle (i.e., weaned calves, feeder cattle, etc.), does the BQA Certification status of the seller influence any of the following? (mark all that apply)

_____No, the BQA Certification status does not influence my purchases
_____Yes, it influences whether or not I intend to purchase the cattle
_____Yes, it influences the price I am willing to pay for the cattle
_____Other (please specify):

30. Please indicate your level of agreement with the following statements:

	Strongly Disagree	Disagree	Neutral	Agree	Strongly Agree
a. The BQA Program helps increase consumer confidence in beef	1	2	3	4	5
b. The BQA Program helps unify the beef industry on topics like animal welfare	1	2	3	4	5
c. All cattle producers should be BQA Certified	1	2	3	4	5
d. I would be interested in participating in a special BQA Certified feeder calf sale/marketing event at a livestock auction market	1	2	3	4	5
e. I would be interested in hosting a BQA Certification training on my operation	1	2	3	4	5
f. Following BQA guidelines is critical to ensuring the safety and end-product quality of beef	1	2	3	4	5
g. Following BQA guidelines is beneficial to the health and well-being of my cattle	1	2	3	4	5
h. The BQA Program is valuable to my operation	1	2	3	4	5
i. Following BQA guidelines improves my operation's profitability	1	2	3	4	5
f. I would support a voluntary contribution to help fund advocacy efforts for the cattle industry in Colorado.	1	2	3	4	5

31. What is your **preferred** method to complete a BQA Certification?

_____In-person
_____On-line training via a self-study platform
_____Other virtual means (webinars, etc.)
_____Other (please specify):

32. Which of the following general sources do you use most often to gain information about BQA and the cattle industry in Colorado? (mark all that apply)

_____Cattle or producer associations
_____Face-to-face cattle producer meetings
_____Friends and neighbors
_____Internet (including social media, fact sheets)
_____Livestock auction market
_____Local university Extension representative
_____Nutritionist
_____Printed publications (magazine, newspaper)
_____State BQA Coordinator or local BQA Trainer
_____Veterinarian
_____Other (please specify):

33. How old are you?

34. What gender do you identify with?

_____Man
_____Woman
_____Non-binary
_____Other
_____Prefer not to answer

35. How many total **mature cows** do you have on your operation (cow/calf and dairy operations)?

_____Less than 50
_____50 to 99
_____100 to 199
_____200 to 499
_____500 to 999
_____1,000 to 4,999
_____5,000 or more

_____N/A

36. How many total head of **calves and/or feeder cattle** do you have in your yearling, stocker, backgrounder, or feedyard operation?

_____Less than 100
_____100 to 999
_____1,000 to 4,999
_____5,000 to 24,999
_____25,000 to 49,999
_____50,000 or more
_____N/A

37. How many years have you been working in the cattle industry?

_____Less than 1 year
_____1-3 years
_____4-10 years
_____11-25 years
_____26-50 years
_____More than 50 years

38. On your cattle operation, which title best describes your primary role? (mark only one)

_____Owner
_____Manager/herdsman
_____Owner and manager/herdsman
_____Hired labor
_____Contract labor
_____Other (please specify):

39. What is the 5-digit zip code where your **primary** cattle operation is located?

40. Is there anything else you would like to share about BQA or the Colorado BQA Program?
